# A four-factor model of psychopathy assessed via neural reinforcement sensitivity theory

**DOI:** 10.1017/pen.2026.10008

**Published:** 2026-07-07

**Authors:** Ella M. Dickison, Phoebe Suat-Hong Neo, Calvin K. Young, Neil McNaughton, Martin Sellbom

**Affiliations:** Department of Psychology, University of Otago, New Zealand

**Keywords:** Anxiety, Attraction, Electroencephalogram, Panic, Psychopathy, Reinforcement sensitivity theory

## Abstract

Reinforcement sensitivity theory (RST) most clearly relates to internalizing disorders. But a weak behavioural inhibition system (BIS as defined by RST) could underlie externalising, in general, and psychopathy in particular (Fowles, 1980). Conventional “rationally derived” RST scales (rRST) are not anchored in neurally defined RST systems (nRST). So, here, we use both rRST and nRST measures to assess psychopathy traits. We operationalised psychopathy via a four-factor model (*affective* | *interpersonal* | *disinhibition* | *boldness*). We operationalised rRST via the Heym, Ferguson & Lawrence (2008) updated version of Carver and White’s (1994) BIS/BAS scales (*BAS | BIS | FFFS)*. We operationalised nRST (goal inhibition system, GIS; goal repulsion system, GRS) via previously validated (Shadli et al., 2021) rhythmic power in the stop signal task (SST) and (goal approach system, GAS) via previously validated ERPs in the doors task. Initial bivariate correlations of psychopathy factors with rRST scales were as expected. We found no significant associations between psychopathy factors and nRST measures. A series of post hoc exploratory repeated measures ANOVAs guarded against non-linearity between psychopathy and nRST constructs. These found that: (1) *Disinhibition* traits might be explained (unexpectedly) by *increased* sensitivity in the GIS (i.e., conflict) and GRS (i.e., repulsion) and *decreased* sensitivity in the GAS (i.e., attraction). (2) *Affective* traits might be explained, as expected, by decreased sensitivity in the GIS and GRS. But an unexpected positive association was also found in the alpha frequency range for the GRS. So, nRST systems (particularly GIS) do not explain psychopathy. rRST scales were more aligned with expectations but were explained via their “rational” basis not RST per se. Unlike internalizing, nRST does not appear strongly related to externalising disorders in general and psychopathy in particular. rRST appears distinct from nRST.

Psychopathy is complex. It is a deviation from normal mental function that impacts the entire personality spectrum. “Psychopaths” commit more violent crimes and have a higher risk of recidivism than other offenders (Douglas et al., 2018). Psychopathy is also associated with drug and alcohol abuse (Ellingson et al., 2018), sexual aggression (Knight & Guay, 2018), physical aggression (Porter et al., 2018), toxic work environments (Mathieu et al., 2020), and violation of public trust (Gao & Raine, 2010; Neo et al., 2018). There is no doubt that psychopathic individuals pose a significant threat to the community and are a public health concern (Reidy et al., 2015).

## 1. The structure of psychopathy

There have been decades of debate as to what psychopathy is (see Miller & Lynam, 2015). We see psychopathy as having a four-factor structure: *Affective | Interpersonal | Disinhibition | Boldness*. This theoretically maps to three previously well-established domains: *Affective | Interpersonal | Behavioural* (e.g., Cooke & Michie, 2001). But we add the construct of *Boldness.* This has emerged from the triarchic psychopathy model (Patrick et al., 2009). In our hands, this four-factor structure emerged from a multi-modal factor analysis on the same population as the current study (Dickison & Sellbom, 2026).

***Affective*** symptoms of psychopathy describe someone as emotionally shallow, socially contemptuous, detached, exploitative, cruel, and lacking in guilt, remorse, and empathy – often referred to as ‘callous-unemotional traits’ and considered to be a central component to psychopathy (e.g., Sellbom & Drislane, 2021).

***Interpersonal*** symptoms of psychopathy describe someone as manipulative, dominant, and deceitful in their interactions with others (e.g., Draycott et al., 2011).

***Disinhibition*** describes an overall externalising proneness, embodying symptoms of antisocial behaviour, impulsivity, sensation-seeking, poor behavioural control, and emotion regulation (Dickison & Sellbom, 2026).

***Boldness*** describes symptoms of fearlessness, social dominance, and self-assurance (Dickison & Sellbom, 2026).

## 2. Previous neural models of psychopathy

Several neurobiological and neurocognitive models of psychopathy have been developed (see Stratton et al., 2015 for review). But it is currently unclear how the proposed model processes manifest as traits in psychopathy.

Most of the literature has taken a top-down approach. Researchers first identify superficial patterns of symptoms of psychopathy and then seek their underlying neurobiological correlates. This has contributed useful information to the understanding of the neurobiological aetiology of psychopathy. But this approach makes it difficult to provide a multi-dimensional account of psychopathy that explains how multiple levels of brain functioning influence psychopathic traits.

Using a bottom-up approach – where an already defined neurobiological model is applied to see if or how it can explain psychopathy – would capture the interplay between neural structures, neurotransmitter systems, and behavioural manifestations. For this we need a detailed existing neuropsychological theory of motivational systems.

## 3. Reinforcement Sensitivity Theory (RST)

Reinforcement Sensitivity Theory (RST) is a neuropsychologically-based theory of systems where their overall sensitivities can be seen as underlying personality factors. RST as a personality theory has been applied widely across psychology domains, including personality, clinical psychology, and neuropsychology. RST was developed from Jeffery Gray’s initial pioneering research, spanning several seminal studies (Gray, 1967, 1969, 1970a, 1970b; Gray & Smith, 1969), that focused on better understanding the intricate biological underpinnings of anxiety, associated behaviours, and their neurology. In particular, Gray linked anxiety (defined via anxiolytic drugs) to a Behavioural Inhibition System (BIS).

Gray (1982) expanded this to a detailed theory of three systems: The Behavioural Approach System (BAS), the BIS, and Fight, Flight, Freeze System (FFFS). The neural elements of the theory were updated by Gray and McNaughton (2000) and then by McNaughton and Gray (2024). The core function of these systems is the *state* control of ongoing behaviour. *Trait* descriptions of RST then derive from the *long-term overall* sensitivities that dispose to behaviours and emotions associated with each of the systems. These sensitivities constitute personality traits (Corr & McNaughton, 2008) and their values determine the specific behaviours expressed by an individual given particular environmental circumstances. For example, at any given level of physical threat, a high trait fear individual acts as though they are closer to the threat than would a low trait threat individual.

From 1970 onwards, the BAS (approach) was seen as a ‘reward’ system. It was associated with sensitivity to the presentation of positive reinforcers or omission of expected negative reinforcers; and linked to personality traits of optimism and impulsivity. The BIS was initially seen as a “punishment” system – sensitive to presentation of negative reinforcers or the omission of expected positive reinforcers. But this needs clarification.

“[Gray] distinguished between a learning-related ‘punishment mechanism for passive avoidance [and] a separate punishment mechanism for organizing the unconditioned response to a punishment. We shall call this the “fight/flight” system’ (Gray, 1971, p. 194; note that ‘punishment’ means three different things within this one quote).”

(McNaughton & Corr, 2019, p. 129).

So “punishment” can control active avoidance, passive avoidance, and escape; or can be linked to linked to traits of fear-proneness or phobic avoidance or panic-proneness. Later, the BIS was clarified as the ‘goal conflict resolution’ system (activated by concurrent opposing approach and avoidance tendencies) controlling passive (as opposed to active) avoidance, arousal, attention, and risk assessment; and linked to high trait anxiety (Corr & McNaughton, 2016).

## 4. “Rational” RST measures (rRST)

Much research investigating the relationship between personality traits and RST systems has used “rational” quasi-lexical approaches, representing the RST systems in self-report measures derived from experimenters’ expectations. We will refer to these rationally-derived measures as rRST. There is a wide range of noticeably different personality questionnaires for rRST (see Corr, 2016 for review), and the rRST field has become muddled in assessment, which may impede the theory’s progress, not only for personality but psychopathology as a whole. Neural RST (nRST) is a multi-faceted theory that is difficult to condense down to rational, self-report scales that acknowledge the complexities involved in the theory and none have been validated via the underlying neurology or via the defining pharmacology. This has caused conflict within the literature and hindered comparison between studies (see Corr, 2016 for review).

Carver and White’s (1994) BIS/BAS scales (C&W BIS/ C&W BAS) are the most commonly and widely used measures of rRST in personality (approaching 10,000 citations according to Google Scholar and 5,000 according to Web of Science). It should be noted that the C&W BIS scale was derived from Carver’s personal concept of “anxiety.” It contains no “behavioural inhibition” items but has 40% worry items. Critically, C&W BIS correlates with internalising disorders in general and is not specific to those disorders selectively sensitive to anxiolytic drugs (Griffith et al., 2010). It shares 80% of its variance with NEO-PI-R *Anxiety* (which in turn shares 96% variance with Penn State *Worry*). It is, therefore, a worry scale that is not specific to Gray’s original neurally-defined BIS.

Heym et al. (2008) updated the use of the Carver and White’s scales in a way of particular relevance to the current study. They retained the original “BIS” items but split these into a BIS-anxiety scale and FFFS-fear scale to better match the neurology of Gray and McNaughton (2000). They retained the original BAS scale (which already consisted of 3 subscales and clearly did not match Gray’s neurology). Researchers using the updated scales to investigate psychopathy have found lower C&W BIS-anxiety and higher BAS scales to be associated with disinhibition-behavioural traits, deficient FFFS-fear and BIS-anxiety were associated with affective-interpersonal traits (Broerman et al., 2014; Hughes et al., 2012; Johnson et al., 2014; Roose et al., 2011).

## 5. Neural RST measures (nRST)

**Figure 1.**
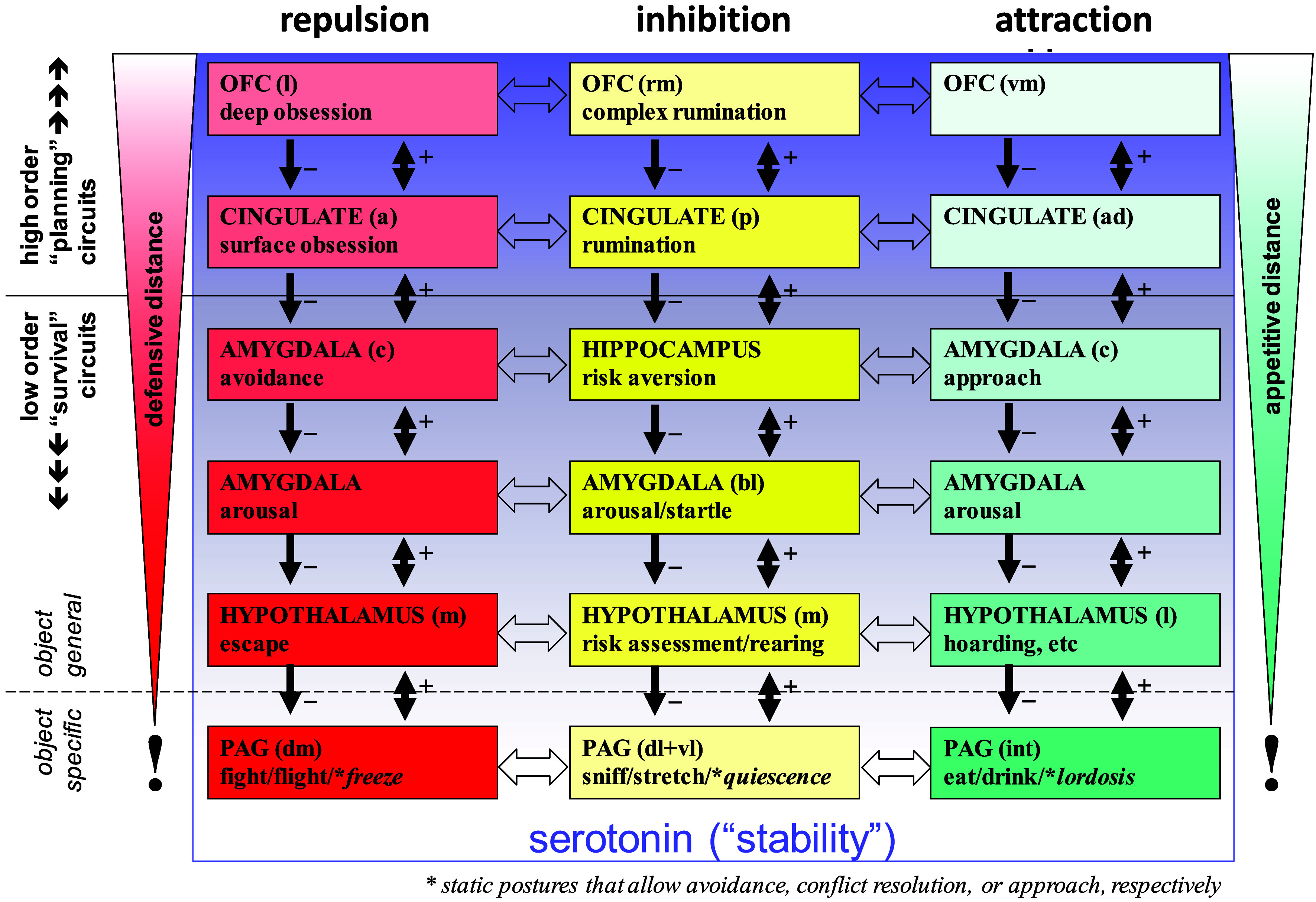
Figure 1 long description. The Interplay Between Micro and Macro Levels in the Brain of the RST Systems. *Note:* Used with permission from McNaughton (2020) and abides by the copyright open policy of Oxford University Press. This figure presents the interplay between micro and macro levels concerning the motivational systems in the brain. repulsion refers to the goal repulsion system (GRS), conflict refers to the goal inhibition system (GIS) and approach refers to the goal attraction system (GAS). These systems are organised hierarchically and outlines the interplay between levels in the brain in response to appetitive and defensive distance of reinforcers. Hormonal compounds, such as benzodiazepine ligands and neuromodulators, interact with the systems. The gradation of purple shading in the figure refers to the capacity of serotonin to shift control from lower to higher levels of the systems.

McNaughton (2020) proposed a new nomenclature for the nRST systems to differentiate between rRST constructs and the neural structure sensitivities of nRST, described in Gray and McNaughton (2000) and further developed in the latest edition of the *state* theory (McNaughton & Gray, 2024). Moreover, he emphasised the importance of employing neurobiological methodologies over rational/lexical measures to accurately capture the intended neurobiological constructs. Because Gray’s original RST is a neurobiological theory, it seems more fitting to explore the links between systems and personality using neural assessment rather than focusing solely on rational constructs based on “postulated roles.” We recognise that there may be practical barriers to this approach, and we need to acknowledge that the rational constructs are helpful in analysis. However, any difference between the questionnaire scales and the neural constructs must be determined and acknowledged. nRST is a neural theory that incorporates multiple levels of the brain, and proposes that each neural system controls multiple behaviours which depend on motivational distance (see Figure 1). This motivational distance depends both on, e.g., the physical level of threat (e.g. distance from a predator) and the trait sensitivity of the individual to threats in general.

The theory has always involved three distinct *neural* systems, which from 2000 onwards were seen as having equivalent contributions. In 2020 they were renamed to match this: the Goal Attraction System (GAS), the Goal Repulsion System (GRS, previously FFFS), and the Goal Inhibition System (GIS). BAS, BIS-anxiety and FFFS-fear scales refer to *rational/lexical descriptions* of approach, passive avoidance, and active avoidance, respectively. In contrast, the GAS, GRS, and GIS, refer to *neural constructs* involving structures that control attraction, repulsion, and goal conflict (defined via anxiolytic drug action), respectively. Repulsion is sensitive to panicolytic drugs but not anxiolytic drugs. We provide neural measures of the trait sensitivities of these nRST systems.

## 6. Psychopathy and RST

There are long-standing theories about psychopathy that have used the concepts of reward (linked to GAS; approach), punishment (linked to GRS; active avoidance), and response inhibition (linked to GIS; passive avoidance/goal conflict). Specifically, psychopathic traits and behaviours have been explained through the out the literature by high sensitivity to reward (e.g., Bjork, Chen & Hommer, 2012; Bjork, Knutson & Hommer, 2008; Bjork, Smith, Chen & Hommer, 2010, 2011; Buckholtz et al., 2010; Engelmann & Tamir, 2009), low sensitivity to punishment (see Patrick, 2018 for review) and difficulties with response inhibition (see Gillespie et al., 2022 for review). The explicit suggestion that a weak BIS was a key element of psychopathy was made many years ago (Fowles, 1980), but despite considerable discussion (McNaughton, pers. comm.) was never accepted by Gray and McNaughton.

In a previous published study (Dickison et al., 2024), we were the first to examine the associations between psychopathy measures and nRST constructs using EEG methodology. The GAS was operationalised through the Gold Bar / Lemon Task (Potts et al., 2006), and the GIS and GRS were operationalised through the Shadli et al. (2015) modification of the stop signal task (SST; Logan et al., 1984). Because the actions of anxiolytic drugs define the GIS, we used the well-established theta-frequency goal-conflict-specific-rhythmicity (GCSR; Neo et al., 2011) to operationalise the GIS and *goal-conflict*. Although there are no direct biomarkers yet established for the GRS and GAS, we used EEG measures of sensitivities that input into each RST system. For the GRS, we used the EEG proxies that measure *outcome conflict* (P. S. H. Neo et al., 2024) due to the GRS outlining active avoidance and response to negative reinforcers. For the GAS, we used dopamine-mediated reward-prediction-error (RPE; Wacker & Smillie, 2015) due to the association between dopamine and engagement with positive reinforcers (DeYoung, 2013).

We found that traits of psychopathic personality disorder may be associated with a dysfunction in the neural processes involved with the GRS and GIS, but there was no evidence of dysfunction involving the GAS. However, the effect sizes of our findings were small, and we observed an unexpected *positive* correlation between affective-interpersonal traits of psychopathy and the GRS. These results required further investigation and replication before any firm conclusions could be derived.

## 7. The current study

The goal of the current study was to further investigate the associations between psychopathy and neural RST, and specifically, to replicate and extend the findings in Dickison et al. (2024) using a different population. We also considered the associations between psychopathy domains and the Heym et al. (2008) adaptation of Carver and White’s (1994) BIS/BAS scales to compare results between studies. As mentioned, there is a well-established literature that assesses psychopathy in terms of Carver and White’s (1994) BIS/BAS scales or other rational scales of RST. See Table 1 for hypothesised correlations.

**Table 1.** Predicted zero-order correlations between the scores of the four-factor model of psychopathy with Carver and White’s (1994) BIS/BAS scales, and EEG measures associated with the RST constructs

	FFFS-fear	BIS-anxiety	BAS
**F1 – Boldness**	−	−	+
**F2 – Disinhibition**	0	−	+
**F3 – Affective**	−	−	0
**F4 – Interpersonal**	−	−	+
	**OCSR (GRS)**	**GCSR (GIS)**	**RewP (GAS)**
**F1 – Boldness**	−	−	+
**F2 – Disinhibition**	0	−	+
**F3 – Affective**	−	−	0
**F4 – Interpersonal**	−	−	+

*Note:* + = positive association hypothesised. − = negative association hypothesised. 0 = no association hypothesised. BIS = behavioural inhibition system. FFFS = fight, flight, freeze system. BAS = behavioural approach system. OCSR, outcome conflict specific rhythmicity (4–7 Hz); GCSR, goal conflict specific rhythmicity (4–7 Hz); RPE, reward prediction error; t2, 70–100 ms; t4, 200–300 ms. GRS = goal repulsion system, GIS – goal inhibition system, GAS = goal attraction system.

For associations with the C&W BIS/BAS scales, we expected positive associations between overall psychopathy and the C&W BAS scale as well as negative associations with the C&W BIS and C&W FFFS scales to align with the findings in Dickison et al., (2024) and previous research findings (e.g., Broerman., 2014; Donahue & Caraballo, 2015; Hughes., 2012; Johnson et al., 2014; Newman et al., 2005; Roose et al., 2011; Ross et al., 2009; Satchell et al., 2018; Uzieblo et al., 2007; Wallace et al., 2009). For associations with the specific psychopathy factors, we anticipated that the C&W BIS/BAS scales would follow the same associations as their neural parallels as reported below. Moreover, we hypothesised that *Boldness* would be negatively associated with the BIS-anxiety and FFFS-fear and positively with the BAS; *Disinhibition* would be positively associated with the BAS and negatively associated with the BIS-anxiety; *Affective* would be negatively associated with both the FFFS-Fear and BIS-anxiety; *Interpersonal* would be positively associated with the BAS, and negatively associated with the BIS-anxiety and FFFS-fear.

In the current study, we operationalised the GIS and GRS in the same way as reported in Dickison et al. (2024); however, we used the doors task (Proudfit, 2015) to operationalise the GAS and elicit *attraction* behaviours. The doors task elicits reward positivity (RewP), which also taps into reward processes definitive of the “dopaminergic” reward circuit (Becker et al., 2014; Carlson et al., 2011; Foti et al., 2011), indicating it is an appropriate input to the GAS.

We hypothesised that the *Boldness* factor would be associated positively with the GAS and negatively with the GIS and GRS. Boldness, within psychopathy, has been associated with personality traits such as extraversion and leadership (see Sleep et al., 2019 for meta-analysis). Aspects of boldness outlining extraversion, leadership, social dominance, and self-assurance describe approach and attraction behaviours prototypical to the GAS (Corr., 2016; Gray & McNaughton, 2000; McNaughton & Gray, 2024). Moreover, the GRS and GIS are neural systems of fear and anxiety, respectively, and boldness has been negatively associated with both constructs (see Lilienfeld et al., 2018 for review).

We hypothesised that the *Disinhibition* factor would be associated positively with GAS and negatively with GIS. The *Disinhibition* factor describes an overall externalising proneness, reflecting antisocial and impulsive aspects of psychopathy. Behavioural aspects of psychopathy are often associated with *higher* levels of anxiety than affective-interpersonal aspects (see Derefinko, 2015 for review) and are characterised by behavioural manifestations representing excessive approach (e.g., impulsivity).

We hypothesised that the *Affective* factor would be associated negatively with both the GIS and GRS. The *Affective* factor represents the traits of psychopathy typically referred to as ‘callous-unemotional’, quintessential for Factor 1 of the PCL-R and primary psychopathy (e.g., Sellbom & Drislane, 2021). As mentioned, researchers have consistently found that affective traits of psychopathy are associated with low levels of anxiety (see Derefinko, 2015 for review).

We hypothesised that the *Interpersonal* factor would be negatively associated with the GRS and GIS and positively with the GAS. Finally, the *Interpersonal* factor describes someone as dominant, manipulative, and deceitful towards others. Cluster analysis studies have found that interpersonal domains of psychopathy often differentiate primary from secondary psychopathy, with higher interpersonal callousness traits more closely aligning with primary psychopathy (Swogger & Kosson, 2007; Vassileva et al., 2005). In addition, primary psychopathy is associated with lower fear *and* anxiety levels compared to secondary psychopathy (Hofmann et al., 2021). Dominance in social interactions with manipulation and deceit reflects excessive approach behaviours reflective of the GAS (Corr & McNaughton, 2019).

## 8. Methods

This study was not preregistered. We report below how we determined our sample size, all data exclusions, all manipulations, and all measures in the study.

### 8.1. Participants

Participants were recruited from a community sample in Dunedin, New Zealand, aimed towards individuals with symptoms of psychopathy as part of a wider project. A total of 250 participants were recruited. In a series of separate advertisements, we asked for people with one or more of the following features: (1) drug, alcohol, or anger management problems, (2) ADHD or impulsivity difficulties, (3) having criminal convictions, (4) having been suspended or expelled from school, (5) with one or more of the following characteristics: charming, good at manipulating or ‘conning’ people, good at taking care of number one, carefree, would do anything for a dare, and adventurous.

Our original grant proposal was for 240 people, based on a power analysis. Since 0.80 power to detect a 0.20 association at *α* = 0.05 requires 191 people; multiple regression analysis with three predictors, minimum *R* ^2^ = 0.05 (based on pilot effect sizes), and 0.80 power, requires 212 participants. A Monte Carlo simulation indicated that, to confirm the latent factor structure of psychopathy, all parameters would be associated with 0.90+ power at *n* = 240.

Of the 250 participants recruited, 19 participants were excluded due to deviant or inconsistent responses on the Minnesota Multiphasic Personality Inventory – 3 (MMPI-3; Ben-Porath & Tellegen, 2020) profiles based on scores on Cannot Say ≥ 15, Combined Response Inconsistency, variable response inconsistency, or true response inconsistency ≥ 80T, and/or infrequent response (F) or infrequent psychopathology responses (Fp) ≥ 100T – with the final sample consisting of 231 participants. Of these, 54% were male, 45% female, and 0.9% were non-binary between 18 and 53 years old. Ethnicity represented a generic New Zealand community sample (65% NZ European, 17% Other European, 14% Māori, 12% Asian, 8% Pacific Islander, 7% Other Descent). The study used different data pools for each measure of RST due to EEG artefacts rendering some data unusable (described in more detail below). The University of Otago Ethics Committee (Health) issued ethical approval (approval number: H20/037).

### 8.2. Measures

#### 8.2.1. Psychopathy measures

We measured psychopathy with a multi-method framework, using self-report and clinician-rated instruments. Clinician-rated measures of psychopathy included the Psychopathy Checklist: Screening Version (PCL:SV; Hart et al., 1995) and the Comprehensive Assessment of Psychopathic Personality: Symptom Rating Scale (CAPP-SRS; Cooke et al., 2012). Four research assistants carried out clinical ratings, so we investigated the consistency of agreement between raters. For a small subsample (*n* = 25), two clinical research assistants completed independent clinical ratings. Inter-rater reliability was calculated via intra-class correlations (ICC) for each clinician-rated measure by using a two-way random model (absolute agreement). Self-report measures of psychopathy used were the Triarchic Psychopathy Measure (TriPM; Patrick, 2010) and the CAPP-Self Report (CAPP-SR; Sellbom et al., 2019).

Table 2 reports the reliability coefficients for each measure. Cronbach’s alpha is generally considered acceptable at 0.7 and good at 0.8 (Nunnally, 1967). The only unacceptably low score is for BIS/BAS-FFFS, but the scale is very brief, and alpha disproportionately penalises short scales.

**Table 2.** Reliability coefficients for scale scores for clinician-rated and self-report measures

Clincian-rated measures		Intra-class correlation	Mean	Standard deviation
PCL:SV	Interpersonal	0.81	0.94	1.18
	Affective	0.82	0.82	1.48
	Lifestyle	0.82	1.15	1.42
	Antisocial	0.71	1.46	1.86
CAPP:SRS	Attachment	0.92	0.74	1.08
	Behavioural	0.92	1.25	0.89
	Cognitive	0.92	1.74	0.93
	Dominance	0.90	0.97	0.89
	Emotional	0.93	1.04	0.95
	Self	0.91	0.93	0.85

Self-report measures		Cronbach’s alpha	Mean	Standard deviation
TriPM	Boldness	0.70	53.02	10.09
	Meanness	0.81	36.41	9.10
	Disinhibition	0.83	44.69	10.65
CAPP-SR	Antagonism/meanness	0.90	1.96	0.45
	Disinhibition	0.82	2.43	0.43
	Fearless grandiosity	0.80	1.99	0.41

RST: Self report		Cronbach’s alpha	Mean	Standard deviation
BIS/BAS scales	BAS	0.84	42.53	5.33
	BIS	0.80	12.13	2.78
	FFFS	0.61	7.97	1.89

*Note:* PCL: SV = Psychopathy Checklist: Screening Version. CAPP: SRS = Comprehensive Assessment of Psychopathic Personality: Symptoms Rating Scale – the current study used the Clinical Interview. TriPM = Triarchic psychopathy measure. CAPP-SR = Comprehensive Assessment of Psychopathic Personality: Self-Report. MMPI-3 = Minnesota Multiphasic Personality Inventory – Third Edition. Intra-class correlations (ICC) are all (*p* ≤ .001).

We operationalised psychopathy via Dickison and Sellbom’s (2026) four-factor model representing factors of affective, interpersonal, disinhibition, and boldness. Dickison and Sellbom’s (2026) model was derived through exploratory structural equation modelling on the psychopathy measures listed above, while controlling for method variance.

#### 8.2.2. RST measures: self-report

**BIS/BAS Scales.** We used Heym et al. (2008) updated Carver and White’s (1994) BIS/BAS scales to measure RST via self-report. The BIS/BAS scales are a 20-item self-report measure whereby a participant scores the degree that a statement is true or false for them on a 4-point Likert scale. Heym et al. (2008) updated version of Carver and White’s (1994) BIS/BAS scales consist of the BIS-anxiety scale (4 items), the FFFS-fear scale (3 items), and the BAS scale (13 items). Subsequent research has confirmed and supported these scales (e.g., Beck et al., 2009; Dissabandara et al., 2012; Poythress et al., 2008).

#### 8.2.3. RST measures: neural

**Figure 2.**
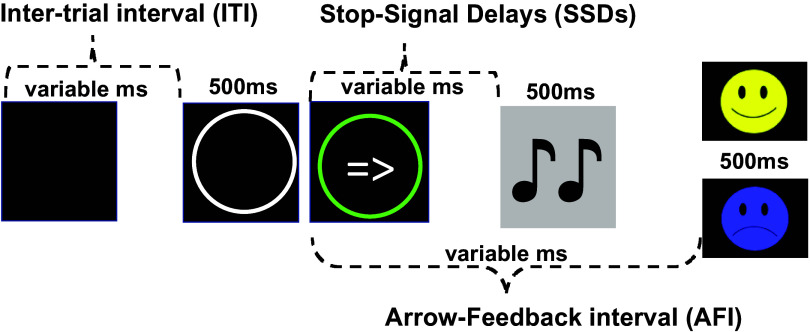
*The Stop Signal Task (SST). The sequence of events in a trial.* This figure is taken from our previous paper, Dickison (2024), as both studies used the same task. The sequence of events in a trial. For a stop trial, the onset of the tone from the time that the arrow is presented (SSDs) are variable. The same event sequence follows for a Go trial, but without the onset of a tone. A smiley is presented for a successful withholding the mouse click, and a frowny is presented for an unsuccessful withholding of the mouse click in a Stop trial. A smiley/frowny is presented for correct/incorrect responses in a Go trial. ms: milliseconds; ITI: 500 ms to 4000 ms; AFI: Go correct = 100 mss; stop fail = 1500 ms; stop correct = 1700 ms. SSDs: the time between when the arrow and tone are presented.

**Stop Signal Task (SST).** ^1^ The current study used the Shadli et al. (2015) modified version of the Aron and Poldrack (2006) SST task. The modified SST task consists of both STOP and GO trials, as shown in Figure 2. During a GO trial, participants are shown a left or right arrow and must respond by making a corresponding left or right mouse click. In a STOP trial, after the arrow appears, a tone signals the participant to withhold their mouse click. STOP trials occur intermittently, with each STOP trial being counterbalanced by three GO trials (the sequence was fixed for all participants). The stop-signal delay (SSD) controls the interval between the presentation of the arrow and the tone. As SSD increases, the likelihood of failing to inhibit the response increases. If participants successfully or unsuccessfully inhibit their response, a happy or frowny face (GRS) provides feedback on the screen. Separate staircases were used to distribute STOP trials with varying delays – short, medium, and long. The short and long staircases adjusted the SSD based on recent reaction times during GO trials, while the medium staircase adjusted the SSD up or down by 30 ms depending on whether the participant stopped successfully, maintaining a balance of 50% correct stopping. GIS was assessed via the conflict-specific difference between the medium trials and the average of short and long. Before starting the test trials, participants completed 30 GO-only practice trials. The main test included 99 STOP and 296 GO trials across three blocks, with no breaks between blocks.

**The Doors Task.** *The Doors Task was developed by Proudfit (2015) to elicit responses to losses and gains to measure reward positivity (RewP). The Doors Task elicits reward prediction (RewP), that occurs in response to stimuli. RewP both taps into reward processes and is associated with the “dopaminergic” reward circuit (Becker et al., 2014; Carlson et al., 2011; Foti et al., 2011) theoretically linked to approach motivation traits (DeYoung, 2013; Neo et al., 2021; Wacker & Smillie, 2015; Zisner & Beauchaine, 2015).*

**Figure 3.**
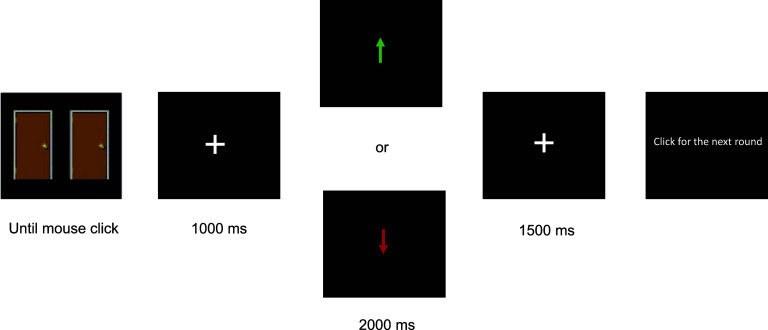
Visual representation of the sequence of events in the Doors Task. *Note:* This figure demonstrates the sequence of events in a trial of the doors task. Green upwards arrow indicates they won money, red downwards arrow indicates that they lost money. ms = milliseconds.

The Doors Task consists of 40 trials. On each trial, the participant is presented with two doors (see Figure 3), requiring them to make a left or right mouse click to select the left or right door, respectively. Once clicked, they are presented with a feedback arrow indicating whether they selected the correct door. An upward arrow indicated a gain of 50 cents, and a downward arrow indicated a loss of 25 cents. Participants were told they would keep the amount accumulated over all trials if there was a net gain; however, a net loss would have no consequences.

The participants were unaware that the monetary outcomes were predetermined, with 20 gain and 20 loss trials counterbalanced and consistent across participants. Participants experienced the same sequence of outcomes regardless of which door they chose and were awarded $5 for the task.

#### 8.2.4. EEG acquisition and processing

All EEG was recorded through an advanced neuro technology (ANT) amplifier at 1,024 Hz with ANT caps and AgCl electrodes. Impedances were kept below 10 KΩ as measured by ANT software (eego). EEG recording was referenced to CPz and sampled at 512 Hz from FP1, FPz, FP2, F7, F3, Fz, F4, F8, T7, T8, C3, Cz, C4, P7, P3, Pz, P4, P8, M1 and M2. The data were processed via Matlab 2019a plugins EEGlab (version 2019_0) and ERPlab (version 7.0.0).

***The SST task.*** The EEG data for the SST task was segmented from the start of the first test trial to the end of the last one and filtered between 1 and 36 Hz using the function *pop_eegfiltnew()*. Eye movement components were identified via independent component analysis (ICA) run through the function *runamica15(spectral power estimates)* on all recording channels. Blink components were automatically removed using *pop_icflag()* with a 90% similarity threshold. Two 1.5-second EEG segments were extracted from each trial, centred at the onset of the Stop-Signal and at the mouse-click response. The *pop_eegthresh()* function removed any epochs where EEG activity exceeded ±70 µV at any sensor. Two consecutive 1-second Hanning windows, overlapping by 0.5 s, were applied to each 1.5-second segment, resulting in two 1-second epochs. The Hanning window cosine function reduced the weighting of edge data within an epoch. Spectral amplitude was extracted from each 1-second epoch using the *fft()* function, squared to obtain spectral power, and log-transformed to normalise the power distribution.

The Hanning window provided spectral power estimates weighted towards the central 0.5 s of each 1-second segment. The second segment of each pair was used to focus on: (1) the 0.5 s following the initial presentation of the Stop-Signal (goal-conflict; GIS), and (2) the 0.5 s after error feedback (GRS). The 0.5 s before these events and after the mouse click were excluded from the analysis. Goal-conflict-related changes in theta rhythmic activity (GIS) have been consistently observed at the right-frontal electrode (F8) (Corr et al., 2016; Neo & McNaughton, 2011; Neo et al., 2011; 2020; Shadli et al., 2021), while outcome-conflict-related changes (GRS) have been seen at the mid-frontal electrode (Fz) (Cohen, 2011; Pasion & Barbosa, 2019; Yeung et al., 2004; Zavala et al., 2016). We, therefore, analysed theta activity at both electrode locations for each system to aid comparisons across systems.

For both goal-conflict (GIS) and outcome-conflict (GRS), the theta power during the corresponding time in a preceding GO trial was subtracted from the theta power in the STOP trial (if the preceding trial was also a STOP trial, the following GO trial was used). For the goal-conflict contrast (GCSR), the average STOP-GO theta power difference for early and late SSDs was subtracted from that of medium SSDs to calculate residual theta power. This residual theta power reflects reactivity to goal-conflict, driven by the balance between stop and go processes when stopping success occurs ∼50% of the time. As stopping is easier in early delay trials and harder in late delay trials, goal-conflict reactivity should peak in medium SSD trials. Trials were excluded if they had too few trials in any response blocks due to excessive EEG noise.

The outcome-conflict (GRS) contrast (OCSR) was calculated from the 0.5-second periods following the mouse-click in both STOP and GO trials. Only GO trials with mouse-clicks and STOP trials with mouse-clicks (indicating outcome conflict) were averaged, so the trials only differed based on whether the response was correct (GO trial) or incorrect (STOP trial). Participants with fewer than 15 trials in each block were excluded.identified three main issues that potentially

**The Doors Task.** The EEG data for the Doors Task were extracted and resampled from 1,024 to 512 Hz. The data were then bandpass filtered (1–36 Hz) using the *pop_eegfiltnew()* function before we used the *runamica15()* function for independent component analysis (ICA; Winkler et al., 2015). Eye component artefacts were automatically removed using the *pop_icflag()* function. Components with >90% similarity to eye components were removed. Following artefact removal, the data was re-referenced to the average of all EEG sensors (i.e. excluding M1 and M2; and after removal of EOG components), and epochs ranging from −1,200 ms to + 1800 ms from the onset of the arrow in the task were extracted. Linear drifts in the data were removed by applying the *detrend()* function to each epoch. Each epoch was segmented from −200 to + 800 ms from the onset of the arrow. Epochs were rejected if their signals were larger than −70/ + 70μV. Participants were also excluded from the analyses if they had less than five trials on average. The total number of participants in the doors task was 210.

### 8.3. Procedure

Participants first completed a battery of self-report questionnaires that were involved in a larger study. The questionnaires were delivered on a computer via Qualtrics and included the BIS/BAS scales, MMPI-3, TriPM, and CAPP-SR, among several others. While the participants completed the battery of self-report measures, the experimenter fitted the EEG cap, applied electro-gel, and reduced the impedance of the EEG cap sufficiently. The participants then completed a series of EEG tasks delivered via computer that included the SST (∼25 minutes) and the doors task (∼5 minutes), among others not used in the current study. After the participants completed the EEG tasks, a research assistant, enrolled in (or completed) a clinical psychology programme, administered a series of structured interviews (CAPP-SRS, PCL-SV, criminal history, substance use modules) under the supervision of a registered clinical psychologist. Participants were reimbursed for their time with a $75 supermarket voucher and up to $10 from winnings in the EEG tasks.

### 8.4. Data analysis

#### 8.4.1. Initial replication

First, zero-order correlations were calculated between each psychopathy factor and the self-report BIS/BAS scales. A correlation of medium effect size (*r* ≥ |.30|) was considered meaningful. We used this threshold due to potential inflation in the correlations from non-construct related reasons (e.g., social desirability) due to the shared method variance across self-report measures. We applied Bonferroni correction to any significant association values adjusting for the total number of possible pair-wise comparisons.

Next, we investigated zero-order correlations between the four-factor psychopathy factor model and neural measures of RST. An a priori alpha level of 0.05 (two-tailed) was used for statistical significance to indicate evidence of an association rather than reliance on a medium-sized effect due to the lack of shared method variance between measures, as well as the magnitude of effect sizes that one typically observes across self-report and neural measures (e.g., Neo, McNaughton, et al., 2024; Neo, Shadli, McNaughton & Sellbom, 2024). If there were any instances where at least two of the psychopathy factors were meaningfully associated with an RST measure, we planned to conduct linear regression analysis to determine which psychopathy factors were uniquely associated with the neural RST measure.

#### 8.4.2. Post hoc analysis

As a result of null findings between psychopathy factors and neural RST constructs, described in the results section below, we decided on a series of unplanned post-hoc analyses for further investigation. The rationales for these post hoc analyses are reported in the results section. The EEG data and processing procedures for the SST and index of GIS (GCSR) and GRS (OCSR) followed the methodology outlined in the Methods section. However, instead of averaging the data over 4–7 Hz frequency range, we extracted the data at individual frequencies within the broader range of 3–13 Hz (theta and alpha).

For the GIS and GRS, a series of repeated measures ANOVAs were conducted with within-subjects factors of electrodes (Fz and F8) and frequency (3–13 Hz) and with a between-subjects factor of psychopathy. For the GAS, the repeated measures ANOVA used within-subjects factor as the time points (t1, t2, t3, t4, t5), and the between-subjects factor as the psychopathy grouping. Psychopathy groups were extracted for each of the four factors separately by splitting participants into groups with low, medium, and high psychopathy scores (relative to the sample), while aiming for an equal number of participants in each group.

### 8.3 Results

#### 8.3.1. Zero-order correlations and regression analysis

Table S1 in the Supplementary Materials presents correlations between all measures. Figure S1 in the Supplementary Materials shows scatterplots of any significant zero-correlations. Here, we will report on the findings that are directly relevant to the research aims. Table 3 shows bivariate correlations between the psychopathy factors and Carver and White’s (1994) BIS/BAS scales. A meaningful positive correlation (≥ |.30|) was observed between Boldness and the BAS scale. Negative correlations with BIS-anxiety were observed for both Boldness and Affective factors. Boldness was also negatively associated with FFFS-fear. To control for the risk of Type I errors due to multiple comparisons, we applied a Bonferroni correction by dividing the significance level by the number of tests conducted (0.05 / 12 = 0.004). These associations all remained significant after correction. Multiple regression analysis between psychopathy factors and the BIS-anxiety scale found that both Boldness and Affective factors contributed equally to the prediction of BIS-anxiety (see Table 4 for multiple regression results), with a suppression effect from the Disinhibition factor (part correlation = 0.00) on the Interpersonal factor (zero-order correlation = −0.21, part correlation = 0.13).

**Table 3.** Zero-order correlations between the scores of the four-factor model of psychopathy and Carver and White’s (1994) BIS/BAS scales

		FFFS-fear	BIS-anxiety	BAS
**F1 – Boldness**	*r*	−0.63* (−0.70, −0.54)	−0.45* (−0.55, −0.34)	0.37* (0.25, −0.47)
	*p*	* < .001*	* < .001*	* < .001*
**F2 – Disinhibition**	*r*	−0.02 (−0.14, 0.11)	**−0.13** (−0.26, −0.00)	**0.02** (−0.11, 0.15)
	*p*	*.82*	*.05*	*.73*
**F3 – Affective**	*r*	**−0.27** (−0.38, −0.14)	−0.46* (−0.56, −0.36)	0.05 (−0.08, 0.17)
	*p*	* < .001*	* < .001*	*.49*
**F4 – Interpersonal**	*r*	**−0.26** (−0.37, −0.13)	**−0.21** (−0.33, −0.08)	**0.20** (0.07, 0.32)
	*p*	* < .001*	*.001*	*.002*

*Note:* Correlations in bold were hypothesised. 95% Confidence intervals are in parenthesis. BIS = Behavioural inhibition system. FFFS = fight, flight, freeze system. BAS = Behavioural approach system. * = Correlation is of at least a medium effect size (0.30 or above).

**Table 4.** Multiple regression analysis with the four psychopathy factors entered to predict Carver and White’s (1994) BIS-anxiety scale. Simple correlations are shown in Table 1

BIS-Anxiety	*R* ^2^	*β*	*p*	Partial	Part
	0.36				
Boldness		−0.43	< .001	−0.44	−0.39
Disinhibition		0.00	.970	0.00	0.00
Affective		−0.44	< .001	−0.42	−0.37
Interpersonal		0.16	.015	−0.21	0.13

*Note: p* = significance. *β* = beta weight. *R* ^2^ = coefficient of determination. Partial = partial correlations (*r*). Part = part correlations (*r*).

Table 5 reports correlations between psychopathy factors and neural measures of RST. There was one meaningful correlation between RewP and Disinhibition, but this finding was no longer significant after applying a Bonferroni correction to account for multiple comparisons. Given the critical *p*-value (.05) and the 16 tests conducted, the *p*-value of .02 was no longer significant after the adjusted significance level (0.05 / 16 = 0.0031). No meaningful correlations were found between any other psychopathy factor and neural RST measures.

**Table 5.** Zero-order correlations between scores on the four-factor model of psychopathy and EEG measures associated with RST constructs

		OCSR (GRS) *n* = 223		GCSR (GIS) *n* = 211		RewP (GAS) *n* = 210	
		F8	Fz	F8	Fz	T2	T4
**F1 – Boldness**	*r*	−0.06 (−0.19, 0.07)	**−0.02** (−0.15, 0.11)	**−0.04** (−0.17, 0.10)	0.03 (−0.10, 0.17)	**−0.03** (−0.16, 0.11)	**0.06** (−0.07, 0.20)
	*p*	.35	.78	.58	.64	.70	.35
**F2 – Disinhibition**	*r*	−0.06 (−0.19, 0.07)	0.08 (−0.05, 0.21)	**0.08** (−0.05, 0.22)	0.06 (−0.07, 0.19)	**−0.10** (−0.23, 0.04)	−0.16* (−0.29, −0.03)
	*p*	.37	.23	.22	.38	.16	.02
**F3 – Affective**	*r*	0.04 (−0.09, 0.17)	**−0.07** (−0.20, 0.06)	**0.02** (−0.11, 0.16)	0.06 (−0.07, 0.20)	−0.11 (−0.24, 0.02)	−0.09 (−0.22, 0.04)
	*p*	.58	.31	.73	.35	.10	.18
**F4 – Interpersonal**	*r*	−0.04 (−0.17, 0.09)	**−0.10** (−0.23, 0.03)	**−0.05** (−0.19, 0.08)	−0.08 (−0.21, 0.05)	**−0.06** (−0.20, 0.07)	**−0.01** (−0.15, 0.12)
	*p*	.52	.14	.44	.24	.35	.86

*Note:* Correlations in bold are hypothesised. 95% Confidence intervals are in parenthesis. OCSR, outcome conflict specific rhythmicity (4–7 Hz); GCSR, goal conflict specific rhythmicity (4–7 Hz); RPE, reward prediction error; t2, 70–100 ms; t4, 200–300 ms. GRS = goal repulsion system, GIS – goal inhibition system, GAS = goal attraction system. * = Correlation is significant at the 0.05 level (2-tailed).

**Limitations of planned analyses.** We identified three main issues that potentially indicate methodological limitations in the initial analysis.

First, by using bivariate correlation analysis, we assumed a linear relationship between psychopathy factors and neural RST indices. If a nonlinear relationship exists, the correlational analysis may fail due to inflation of the error component by the non-linearity.

Second, we averaged the data over 4–7 Hz for correlation analysis with the SST task and the GIS and GRS investigation. Averaging over 4-7 Hz may dilute the specificity of the analysis and, in turn, lose variations of patterns specific to particular frequencies. Indeed, research by McNaughton et al. (2013) found GCSR (the EEG biomarker for the GIS) within the range of 5–10 Hz with a peak at approximately ∼8 Hz. In addition, while there is evidence that theta is relevant within personality types with high extraversion and low neuroticism, personality traits, such as those involving behavioural inhibition (e.g., trait anxiety), are not linked exclusively to theta but also delta, alpha, and gamma (see Mitchell et al., 2008 for review).

Third, we limited the analysis within the Doors Task when investigating the GAS to time 2 (t2; 70–100 ms) and time 4 (t4; 200–300 ms) based on previous findings indicating that it is at these times where fundamental phasic dopamine components peak (Neo et al., 2021; Redgrave & Gurney, 2006; Schultz, 2016; Schultz et al., 2017). Additionally, Neo et al. (2021) reported positive correlations between Carver and White’s (1994) BAS scale and t2 and t4 in the same population of participants as in Dickison et al. (2024). However, key differences between the populations of interest may influence peaks within these time frames to vary between populations. Dopamine release patterns can vary across individuals and populations due to several factors such as genetic differences, neurobiological diversity, and environmental influences; therefore, using time points identified in one population may not generalise to another (Marinelli & McCutcheon, 2014), especially without several replications of the same finding.

In the post-hoc analysis, we explored nonlinear relationships across diverse frequencies for GIS and GRS and various times for GAS. This analysis will help to understand better the relationship between psychopathy factors and neural RST constructs.

#### 8.3.2. Repeated measures ANOVA

**Figure 4.**
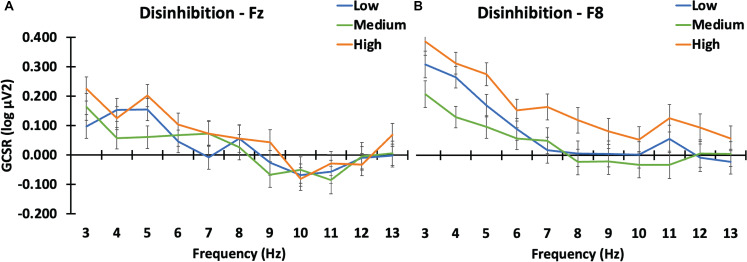
*EEG power of disinhibition groups on GCSR at Fz and F8*. *Note:* The Y axis represents the EEG power (log µV^2^) for the GIS (GCSR) measure. Disinhibition groups are: low (blue); medium (green; and high (orange). The X-axis is the frequency (Hz). **A**. Findings at Fz. **B**. findings at F8. The F8 group difference is significant (see text). Error bars represent standard error.

**Goal Inhibition System (GIS) – GCSR.** Figure 4 presents the EEG power (log µV^2^) for the disinhibition group on GCSR at electrodes Fz and F8. A significant main between-subjects effect of the disinhibition group was observed (F(2, 209) = 4.12, p = .02), most evident at electrode F8 (electrode × group F(2, 209) = 3.51, p = .03). The high disinhibition group elicited higher levels of GCSR than the medium and low disinhibition groups in the 5–7 Hz frequency range (frequency [order 8] x group F(2, 209) = 3.32, p = .04).

**Figure 5.**
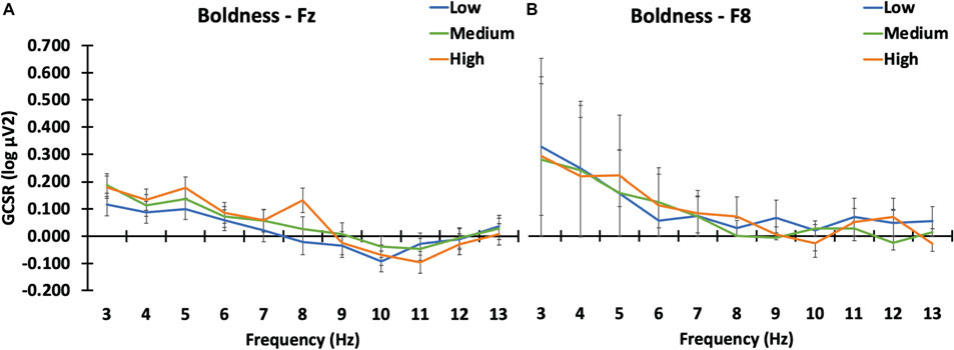
*EEG power of boldness groups on GCSR at Fz and F8*. *Note*: The Y axis represents the EEG power (log µV^2^) for the GIS (GCSR) measure. Boldness groups are: low (blue); medium (green; and high (orange). The X-axis is the frequency (Hz). **A**. Findings at Fz. **B**. findings at F8. Error bars represent standard error.

**Figure 6.**
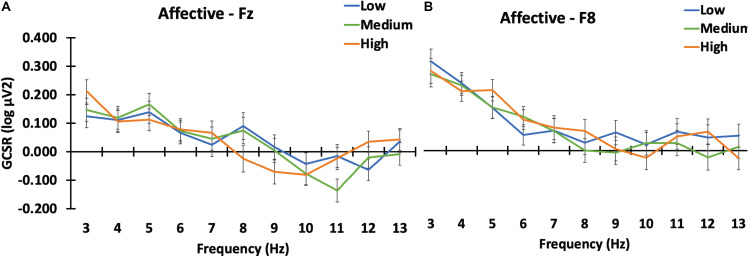
EEG power of affective groups on GCSR at Fz and F8. *Note:* The Y axis represents the EEG power (log µV^2^) for the GIS (GCSR) measure. Affective groups are: low (blue); medium (green; and high (orange). The X-axis is the frequency (Hz). **A**. Findings at Fz. **B**. findings at F8. Error bars represent standard error.

**Figure 7.**
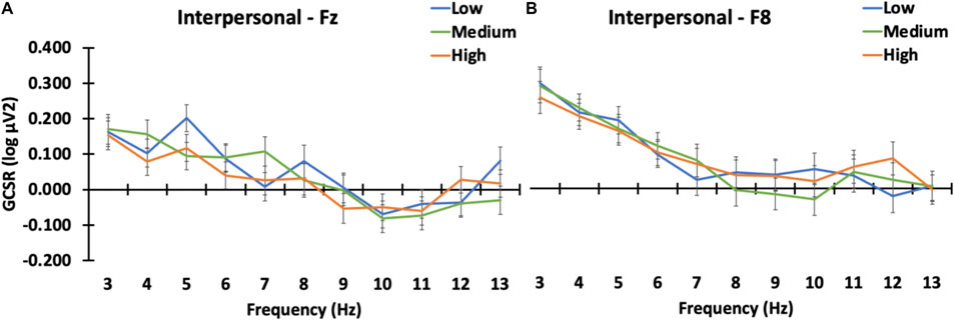
EEG power of Interpersonal Groups on GCSR at Fz and F8. *Note:* The Y axis represents the EEG power (log µV^2^) for the GIS (GCSR) measure. Interpersonal groups are: low (blue); medium (green; and high (orange). The X-axis is the frequency (Hz). **A**. Findings at Fz. **B**. findings at F8. Error bars represent standard error.

Figures 5–7 present the EEG power (log µV^2^) for boldness, affective, and interpersonal groups on GCSR at electrodes Fz and F8. No group or group × frequency differences were statistically significant.

**Figure 8.**
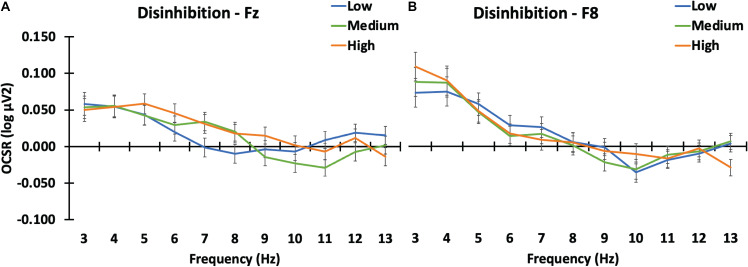
EEG power of disinhibition groups on OCSR at Fz and F8. *Note:* The Y axis represents the EEG power (log µV^2^) for the GRS (OCSR) measure. Disinhibition groups are: low (blue); medium (green; and high (orange). The X-axis is the frequency (Hz). **A**. Findings at Fz. **B**. findings at F8. Error bars represent standard error.

**Figure 9.**
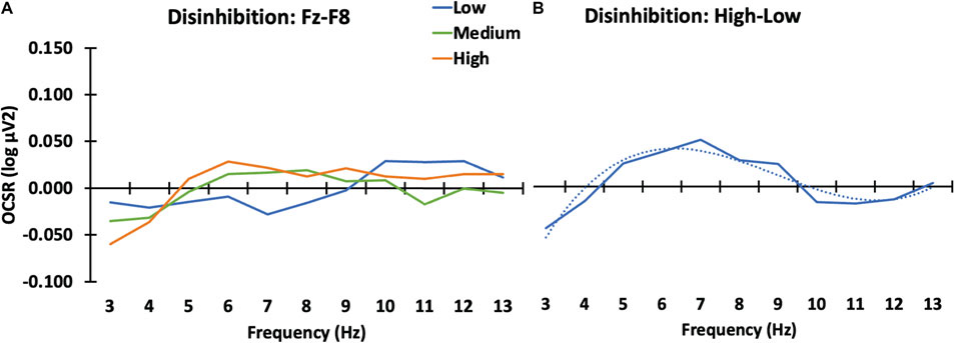
*Interaction effects for disinhibition groups on OCSR*. *Note:* The Y axis represents the EEG power (log µV^2^) for the GRS (OCSR) measure. Disinhibition groups are: low (blue); medium (green; and high (orange). The X-axis is the frequency (Hz). **A**. Findings when the electrode interaction effect is isolated from subtracting Fz scores from F8 scores. **B**. Findings when the group-level interaction effect is isolated from subtracting the low group scores from the high group scores.

**Goal Repulsion System (GRS) – OCSR.** Figure 8 presents the EEG power (log µV^2^) for the disinhibition group on OCSR at electrodes Fz and F8. The high and medium disinhibition groups were significantly higher on OCSR than the low group across 5–8 Hz at electrode Fz but not F8 (group × electrode × frequency[cubic], F(2, 221) = 4.91, p = .01). See figure 9 for the interaction effects for disinhibition groups on OCSR.

**Figure 10.**
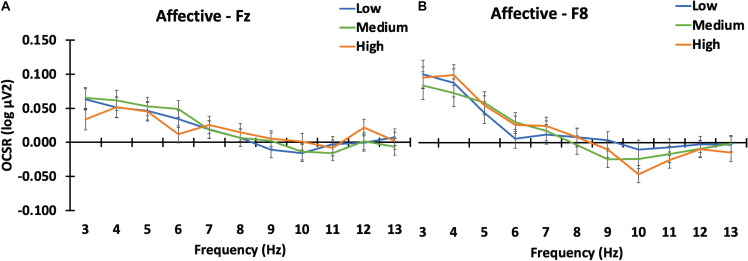
EEG power of affective groups on OCSR at Fz and F8. *Note:* The Y axis represents the EEG power (log µV^2^) for the GRS (OCSR) measure. Affective groups are: low (blue); medium (green; and high (orange). The X-axis is the frequency (Hz). **A**. Findings at Fz. **B**. findings at F8. Error bars represent standard error.

**Figure 11.**
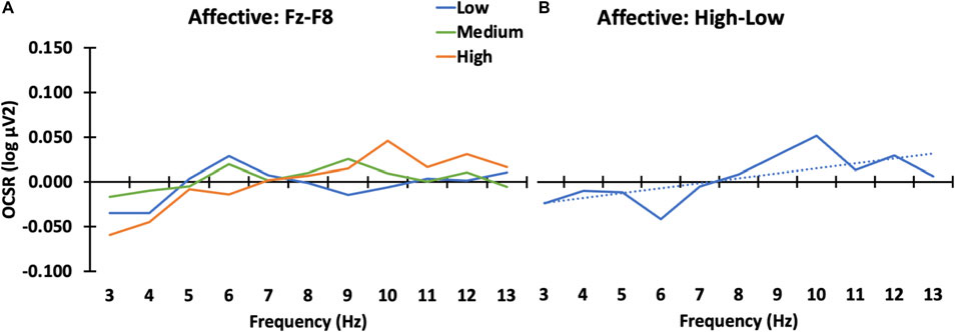
Interaction effects for affective groups on OCSR. *Note:* The Y axis represents the EEG power (log µV^2^) for the GRS (OCSR) measure. Affective groups are: low (blue); medium (green; and high (orange). The X-axis is the frequency (Hz). **A**. Findings when the electrode interaction effect is isolated from subtracting Fz scores from F8 scores. **B**. Findings when the group-level interaction effect is isolated from subtracting the low group scores from the high group scores.

Figure 10 presents the EEG power (log µV^2^) for the affective group on OCSR at electrodes Fz and F8. There was a significant effect of electrode, frequency, and affective group (group × electrode × frequency[linear], *F*(2, 221) = 4.27, *p* = .02). When we isolated the interaction by subtracting the EEG power of F8 from Fz and outlined the difference between the high and low groups (see Figure 11), the difference between the high and low affective groups followed an upward trend as the frequency increased. The high affective group was lower than the medium and low affective groups at lower frequencies (6 Hz), but higher at higher frequencies (10 Hz).

**Figure 12.**
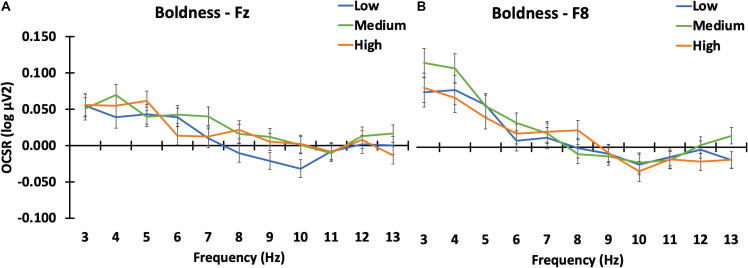
EEG power of boldness groups on OCSR at Fz and F8. *Note:* The Y axis represents the EEG power (log µV^2^) for the GRS (OCSR) measure. Boldness groups are: low (blue); medium (green; and high (orange). The X-axis is the frequency (Hz). **A**. Findings at Fz. **B**. findings at F8. Error bars represent standard error.

**Figure 13.**
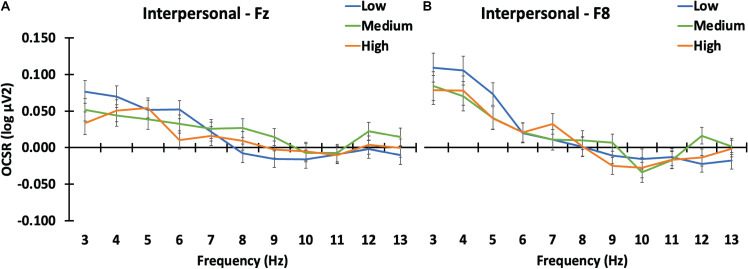
EEG power of interpersonal groups on OCSR at Fz and F8. *Note:* The Y axis represents the EEG power (log µV^2^) for the GRS (OCSR) measure. Interpersonal groups are: low (blue); medium (green; and high (orange). The X-axis is the frequency (Hz). **A**. Findings at Fz. **B**. findings at F8. Error bars represent standard error.

Figures 12 and 13 present the EEG power (log µV^2^) for the boldness and interpersonal groups on OCSR at electrodes Fz and F8. There were no statistically significant findings for differences in the EEG power for boldness and interpersonal groups on OCSR.

**Figure 14.**
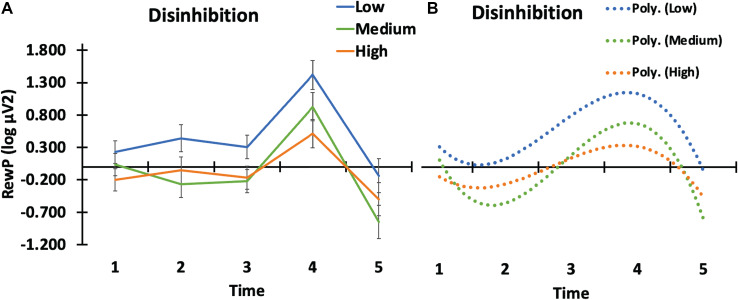
Figure 14 long description. *EEG power of disinhibition group on RewP*. *Note:* The Y axis represents the EEG (log µV^2^) for the measure of the GAS – reward positivity (RewP). The X axis presents the time points (ms): 1 = 0–70; 2 = 70–100; 3 = 100–200; 4 = 200–300; 5 = 300–400. Disinhibition groups are: low (blue); medium (green; and high (orange). Poly. = polynomial. *A* = findings for disinhibition group on EEG measure for GAS. *B* = polynomial trend for each disinhibition group: low (blue); medium (green); and high (orange). Error bars represent standard error.

**Figure 15.**
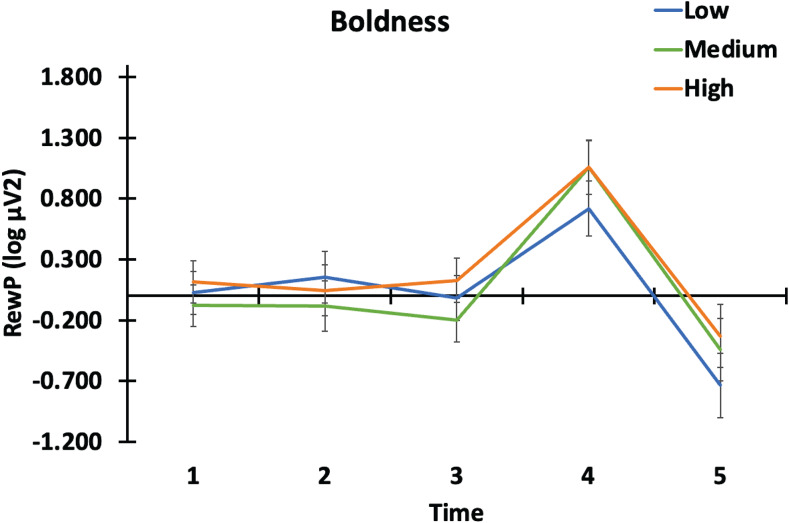
*EEG power of boldness group on RewP*. *Note:* The Y axis represents the EEG (log µV^2^) for the measure of the GAS – reward positivity (RewP). The X axis presents the time points (ms): 1 = 0–70; 2 = 70–100; 3 = 100–200; 4 = 200–300; 5 = 300–400. Boldness groups are: low (blue); medium (green; and high (orange). Error bars represent standard error.

**Figure 16.**
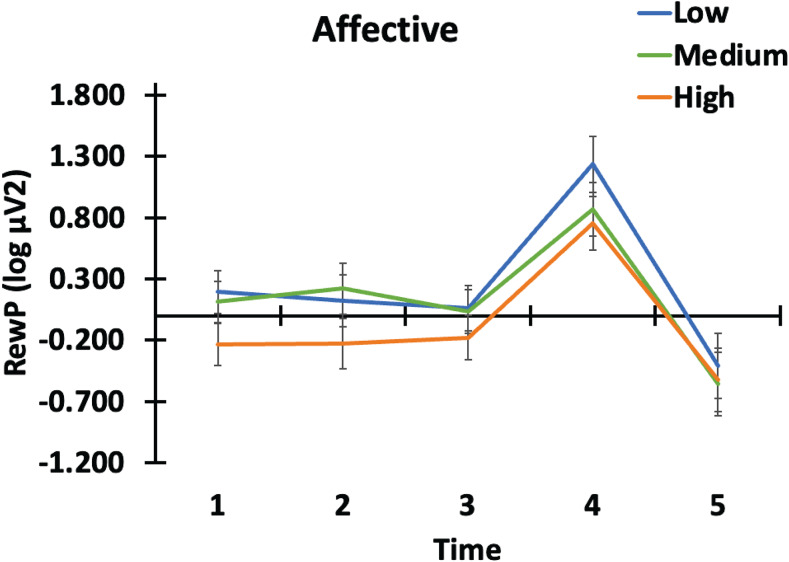
EEG power of affective group on RewP. *Note:* the Y axis represents the EEG (log µV^2^) for the measure of the GAS – reward positivity (RewP). The X axis presents the time points (ms): 1 = 0–70; 2 = 70–100; 3 = 100–200; 4 = 200–300; 5 = 300–400. Affective groups are: low (blue); medium (green; and high (orange). Error bars represent standard error.

**Figure 17.**
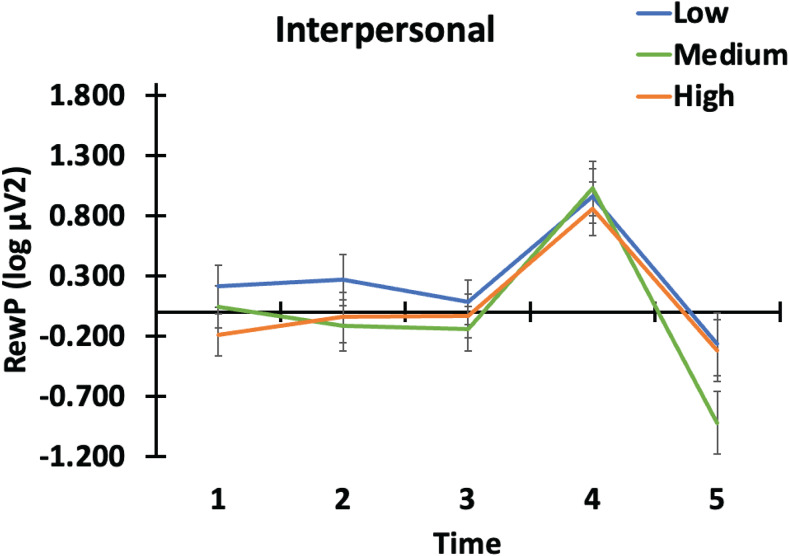
EEG power of interpersonal group on rewP. *Note:* The Y axis represents the EEG (log µV^2^) for the measure of the GAS – reward positivity (RewP). The X axis presents the time points (ms): 1 = 0–70; 2 = 70–100; 3 = 100–200; 4 = 200–300; 5 = 300–400. Interpersonal groups are: low (blue); medium (green; and high (orange). Error bars represent standard error.

**Goal Attraction System (GAS) – RewP.** There was a clear RewP peak at time 4 for all groups for all psychopathy factors. Figure 14 reports the EEG power (log µV^2^) for the disinhibition group on RewP. The low disinhibition group exhibited significantly higher levels of RewP, particularly at times 2 and 4, than the medium and high groups (group × time[cubic] F(2, 211) = 3.23, p = .04). Figures 15–17 display the EEG power (log µV^2^) for boldness, affective, and interpersonal groups on RewP, respectively. Despite sharing the common time 4 peak, none showed any significant group differences.

### 8.4 Discussion

#### 8.4.1. Initial replication

The goal of the current study was to extend the findings from Dickison et al., (2024) and continue investigating the associations between psychopathy factors and RST constructs through traditional rational and novel neural methods. Results from the initial replication mainly were as expected for associations between psychopathy factors and Carver and White’s (1994) BIS/BAS scales and exhibited a similar pattern to that found by Dickison et al., (2024). *Boldness* was negatively associated with FFFS-fear, BIS-anxiety, and positively related to the BAS, and the *Affective* scale was negatively associated with BIS-anxiety. Multiple regression analyses indicated that both *Boldness* and *Affective* contributed equally to predicting negative associations with BIS-anxiety.

However, in the attempt to replicate the results for associations between psychopathy variables and neural RST, we observed null results that were inconsistent with our initial significant findings in Dickison et al., (2024). As noted in Dickison et al., (2024), effect size magnitudes in the initial study were small, with a lack of Bonferroni correction. Therefore, the null findings in a new sample are not entirely dissimilar but require further investigation.

We identified three key methodological limitations in the initial analysis of the current study. First, bivariate correlation analysis assumed a linear relationship between psychopathy factors and neural RST indices, potentially over-simplifying the data. Second, averaging data over 4–7 Hz for EEG analysis may have diluted the specificity of findings, overlooking variations specific to different frequency bands. Third, limiting analysis in the Doors Task to specific time frames for investigating the GAS may not generalise across populations due to individual differences in dopamine release patterns. To address these limitations, we assessed nonlinear relationships between psychopathy and neural RST across a wider range of frequencies and times through repeated measures ANOVA.

#### 8.4.2. Post hoc analysis

For the GIS (GCSR, goal conflict), GCSR levels were higher at electrode F8 than Fz for all psychopathy factors. This is not surprising as research has indicated that GCSR is found at electrode F8 (Corr & McNaughton, 2016; Neo & McNaughton, 2011; Neo et al., 2011; 2020; Shadli et al., 2021). Moreover, the results indicated that individuals higher on the disinhibition factor demonstrated higher levels of GCSR at F8 than individuals with medium or low disinhibition. This non-linear group relationship will have diluted correlations in our initial planned analysis. The findings for the GRS (OCSR, outcome conflict) indicated that individuals with high disinhibition scores elicited higher mean OCSR than medium and low groups at Fz. Individuals with higher affective scores demonstrated significantly lower levels of OCSR at 6 Hz compared to medium and low affective groups, but higher at 10 Hz. These results were observed at Fz, which is to be expected for OCSR (Cohen, 2011; Pasion & Barbosa, 2019; Yeung et al., 2004; Zavala et al., 2016).

Results for the GAS (RewP, attraction) found a significant effect of time, with the highest response at time 4 for all psychopathy factors. There was a significant difference between disinhibition groups on RewP at time 4, with the low disinhibition group eliciting higher levels of RewP than the medium and high disinhibition groupings. The finding of higher disinhibition group scores with higher levels of both GIS and GRS and higher affective scores with lower levels of GRS was as hypothesised in the original analysis. However, the finding of lower disinhibition scores with higher levels of RewP was not expected and requires further investigation.

The following conclusions can be made when comparing the post-hoc findings to the limitations observed in the initial analysis. The first limitation identified in the initial analysis was assuming a linear relationship between variables. In cases where no significant differences between the psychopathy factor group mean on the neural RST measure were found, the observed variations between groups were minimal. Additionally, in cases where there were differences between group means on neural RST measures, this relationship was rarely linear and typically indicated a quadratic or cubic finding. Therefore, by initially assuming a linear relationship, we failed to detect potential relationships between psychopathy and neural RST measures. The second limitation identified was averaging over 4–7 Hz for the GRS and GIS. In cases where there were significant differences between groups on neural RST measures, this included frequencies outside of the 4–7 Hz frequency range. Additionally, this relationship did not necessarily follow a linear trend along frequencies. Therefore, by initially averaging over 4–7 Hz for GIS and GRS, the specificity of the analysis was diluted and, in turn, lost variations of patterns specific to frequencies. Finally, for the third limitation of time specificity with GAS, the results of psychopathy groups on RewP showed a clear and significant interaction at time 4, consistent with expectations. However, peaks at this point did not appear linear across psychopathy group scores. Altogether, post-hoc analysis has provided valuable insights into the possible relationship between psychopathy factors and neural RST and has indicated that the lack of associations in the initial linear correlation analysis was likely due to a lack of linear effect rather than a lack of statistical power.

#### 8.4.3. Interpretation of findings

While we found some significant results in the post-hoc repeated measures ANOVA analysis, most psychopathy factors on each RST system did not elicit significant differences between groups and require further investigation and consideration. However, individuals with higher disinhibition scores exhibited elevated GIS (GCSR, conflict) scores compared to medium and low groups, suggesting a positive association between disinhibition and GCSR, aligning with the initial hypotheses due to the association between anxiety and behavioural traits related to secondary psychopathy (see Derefinko, 2015 for review) but antithetical to the suggestion that a weak BIS is the basis of disinhibition (Fowles, 1980).

We had not hypothesised disinhibition to be associated with GRS (OCSR, fear) in the initial correlational analysis; but high and medium disinhibition groups elicited greater OCSR than the low group. This finding aligns with some literature indicating that secondary psychopathy is associated with ‘adequate’ or more elevated levels of fear compared to primary psychopathy (Corr, 2010; Hofmann et al., 2021). In addition, we found a non-linear relationship between the high affective group and OCSR, with the high group eliciting greater neural signal at 10 Hz, but lower at 6 Hz, when compared to the medium and low groups.

The finding of the low disinhibition group having higher levels of GAS (RewP) compared to the medium and high disinhibition groups was unexpected and did not align with the initial hypotheses with the GAS. However, the association between dopamine expression and externalising disorders, such as ADHD, is complicated. Notably, methylphenidate increases catecholamines including dopamine while reducing disinhibition in ADHD (Vaidya et al., 1998). However, it is not clear how this may present in psychopathy with further investigation is warranted.

#### 8.4.4. Limitations of post-hoc analysis

While the post-hoc analysis provided some insights into the relationship between psychopathy and neural RST, this was not without limitations. We created psychopathy groups, and while this helped investigate nonlinear relationships between psychopathy and neural RST measures, it introduced range restriction within the post hoc analyses.

One methodological limitation is the sample size. While data were collected over three years, and we successfully recruited 250 participants before individual data were excluded in the data processing procedures, the findings are likely underpowered given the small effect size estimates needing detection (Schönbrodt & Perugini, 2013). However, increasing sample size has practical limitations due to the time taken to administer testing to each participant and the materials needed. Compared to other studies investigating psychopathy through EEG methods (see Clark, Bontemps, Batky, Watts & Salekin, 2019 for review), we have collected a relatively large sample. Moreover, given the effect sizes that are typically found in psychopathy research using EEG methods (e.g., Calzada-Reyes et al., 2013; Clark et al., 2019; 2022), the presentation of these findings is valuable as they enhance our understanding of neurobiological mechanisms underlying psychopathy domains. These findings could be beneficial for future pharmacological and other intervention studies.

A further limitation in the ad-hoc analysis of the present study is the polychotomisation of continuous variables into three approximately equal-sized groups for each psychopathy factor relative to the sample. While this approach allows us to consider possible non-linear associations, it reduces variability and restricts range within groups.

It is not surprising that EEG studies in psychopathy typically yield low effect sizes. Specifically, achieving reliability in EEG studies is challenging due to the complexity of capturing dynamic brain activity (Lopes da Silva, 2022). While EEG methods are a powerful tool for investigating neural dynamics due to the relatively low costs involved and non-invasive techniques, they have poor spatial resolution compared to other neuroimaging techniques and primarily capture surface brain activity, which makes it difficult to localise signals to deeper structures in the brain. EEG signals are susceptible to artefacts, both from the participant’s movements as well as external environmental noise resulting in loss of data (which may not be random). In addition, EEG data can vary from individual to individual due to anatomical differences, with limited diagnostic utility (Michel & Brunet, 2019). Finally, the challenge measures used here will have produced results that have state variations superimposed as error variance on trait variations.

#### 8.4.5. Theoretical implications

RST is a dynamic neural theory spanning multiple levels (both macro and micro) of the brain. Specifically, each neural system controls multiple behaviours that tap into different levels and processes depending on motivational distance. With frontal recording our results will have tapped into only the cortical and not subcortical parts of the RST systems.

For example, the GRS controls many different behaviours ranging from flight, fight, freeze behaviours when a threat is proximal to anticipation and repulsion when the threat is distal to pre-emptive planning when it is far in the future. Further, neurotransmitters can interact with the GRS differently at each level (McNaughton, 2020) with serotonin having opposite functional effects on proximal and distal threat (Graeff & Zangrossi, 2010), essentially shifting from fast to slow thinking processes (Carver et al., 2008; Kahneman & Patrick, 2011). Likewise, the GAS can output many different behaviours ranging from consummatory when the reinforcer is proximal to approach when the reinforcer is more distal to extensive goal-subgoal planning when it is far in the future. The GIS outputs risk assessment and behavioural inhibition when the conflict is proximal and rumination when the conflict is distal. All three systems also act to increase arousal and attention when the reinforcer is strong, with neurotransmitters having differential impacts on each level of activation for each neural RST system (McNaughton, 2020). These effects of motivational distance must be taken into account when interpreting links with psychopathy.

It also remains unclear how effectively and comprehensively each of the EEG measures used here represents the *overall sensitivity* of each neural RST system. Our measures are only of the more rostral components of the system. An additional complication is that EEG measures (or any task-specific measure) will include both state and trait components. Where there is strong state variation superimposed on the trait component the result will have a high level of error from the trait point of view – and it is traits that the specific questionnaires we used are designed to capture.

Conversely, the psychopathic domains may be explained by dysfunction in *components* of a neural RST system that the EEG measures of *overall* sensitivity do not assess. For example, while a high score on a global GRS measure would predict higher levels of panic behaviour, some panic disorder is likely to be due to specific problems with the periaqueductal grey, independent of the rest of the GRS (Dantendorfer, Amering, et al., 1995; Dantendorfer, Windhaber & Maierhofer, 1995). We would not expect our EEG measures to capture such subcortical processes.

Finally, our results may simply indicate that RST may not be the most effective means to conceptualise and explain psychopathy domains. RST is a theory of personality derived from the state theory of the neuropsychology of anxiety (McNaughton & Gray, 2024). While the theory is robust in explaining states and internalising disorders, translation to externalising and psychopathy may not be as clear as we initially expected it to be. Within the Big 5 (e.g., DeYoung, 2015), the RST personality traits would be facets (or perhaps, with GRS, the aspect of withdrawal). With 10 aspects, each with multiple facets, there are many non-RST traits that could be fundamental to psychopathy that we do not assess through RST. Therefore, RST may not be the most appropriate personality theory to use to better understand psychopathy.

#### 8.4.6. Conclusion

The current study provides some evidence for associations between psychopathy traits and neural deficits in goal conflict, outcome conflict, and attraction. Moreover, high disinhibition in psychopathy may be explained by elevated goal-conflict and outcome-conflict. High affective scores may be attributed to dysfunction in outcome-conflict at certain frequencies. While not expected, the results also indicated that high disinhibition in psychopathy might be associated with low attraction. However, discrepancies between findings challenge the theoretical and methodological approach taken to better understand psychopathy.

## Supporting information

10.1017/pen.2026.10008.sm001 Dickison et al. supplementary material Dickison et al. supplementary material

## Footnotes

1
The EEG processing of the SST was performed identically as reported in our previous paper (Dickison et al., 2024). The current method text is adapted from this original.

## Supplementary material

The supplementary material for this article can be found at https://doi.org/10.1017/pen.2026.10008.

## Figure 1. Long description

A diagram of the brain’s response systems. The diagram is divided into three main columns labeled repulsion, inhibition, and attraction, and four rows labeled high order planning circuits, low order survival circuits, object general, and object specific. Each section contains labeled brain regions with their associated behaviors. The repulsion column includes regions like the orbitofrontal cortex (OFC) and amygdala, associated with deep obsession and avoidance. The conflict column includes regions like the hippocampus and cingulate, associated with risk aversion and rumination. The approach column includes regions like the OFC and amygdala, associated with approach and arousal. Arrows indicate the directional flow and interactions between these regions. The diagram also includes a gradient bar at the bottom labeled serotonin stability, indicating the role of serotonin in modulating these behaviors.

Navigate back to Figure 1..

## Figure 14. Long description

Two line graphs depict EEG power of disinhibition groups over time. Panel A: A line graph shows EEG power (RewP in log microvolts) on the vertical axis and time on the horizontal axis. The graph includes three lines representing low, medium, and high disinhibition levels. The low disinhibition line peaks at time 4, while the medium and high disinhibition lines show smaller peaks around the same time. Panel B: Another line graph shows polynomial fits for low, medium, and high disinhibition levels over time. The polynomial lines for low, medium, and high disinhibition levels show different trends, with the low disinhibition line peaking higher and later than the medium and high disinhibition lines.

Navigate back to Figure 14..
