# Supplementary material for: A four-factor model of psychopathy assessed via neural reinforcement sensitivity theory
Source: Personal Neurosci. 2026 Jul 7;9:e4. doi: 10.1017/pen.2026.10008 (PMC13370198; doi:10.1017/pen.2026.10008)
Supplement: Dickison et al. supplementary material [file S251398862610008Xsup001.pdf]

Exploring Associations of Psychopathy with Neural Reinforcement Sensitivity Theory Constructs [Supplementary file]

Ella M. Dickison, Phoebe S-H Neo, Calvin K. Young, Neil McNaughton, & Martin Sellbom

Department of Psychology, University of Otago, Dunedin, New Zealand

Contents

Table S1 2
Figure S1 3

Table S1

Zero-order Correlations Between all Measures

Measures		F1 – Boldness	F2 – Disinhibition	F3 – Affective	F4 – Interpersonal	FFFS-fear	BIS-anxiety	BAS	OCSR (GRS) n = 223		GCSR (GIS n = 211		RewP (GAS) n = 210	
									F8	Fz	F8	Fz	T2	T4
**F1 – Boldness**		-												
**F2 – Disinhibition**		-.07	-											
**F3 – Affective**		.21**	.34**	-										
**F4 – Interpersonal**		.43**	-.06	.41**	-									
**FFFS-fear**		-.63#	-.02	-.27	-.26	-								
**BIS-anxiety**		-.45#	-.13	-.46#	-.21	.53**	-							
**BAS**		.37#	.02	.05	.20	-.12	.04	-						
**OCSR (GRS) n = 223**	F8	-.06	-.06	.04	-.04	.07	.10	-.05	-					
	Fz	-.02	.08	-.07	-.10	.10	.08	.01	.66**	-				
**GCSR (GIS) n = 211**	F8	-.04	.08	.02	-.05	.02	.03	-.08	.19**	.08	-			
	Fz	.03	.06	.06	-.08	-.05	-.07	-.08	.20**	.10	.46**	-		
**RewP (GAS) n = 210**	T2	-.03	-.10	-.11	-.06	-.03	.06	-.09	-.04	.03	-.10	-.02	-	
	T4	.06	-.16	-.09	-.01	.02	.13	.03	.03	.01	.01	.05	.42**	-

Note: ** = Correlation is significant at the .01 level (2-tailed). * = Correlation is significant at the .05 level (2-tailed). # = Correlation is of at least a medium effect size (.30 or above). F = Factor. FFFS = Fight, Flight Freeze Scale. BIS = Behavioural Inhibition Scale. BAS = Behavioural Approach Scale. OCSR = outcome specific rhythmicity. GCSR = goal conflict specific rhythmicity. RPE = reward prediction error. GRS = Goal Repulsion System. GIS = Goal Inhibition System. GAS = Goal Attraction System.

Figure S1

Scatterplots of significant correlations from zero-order correlational analyses between psychopathy factors and nRST and rRST scales.

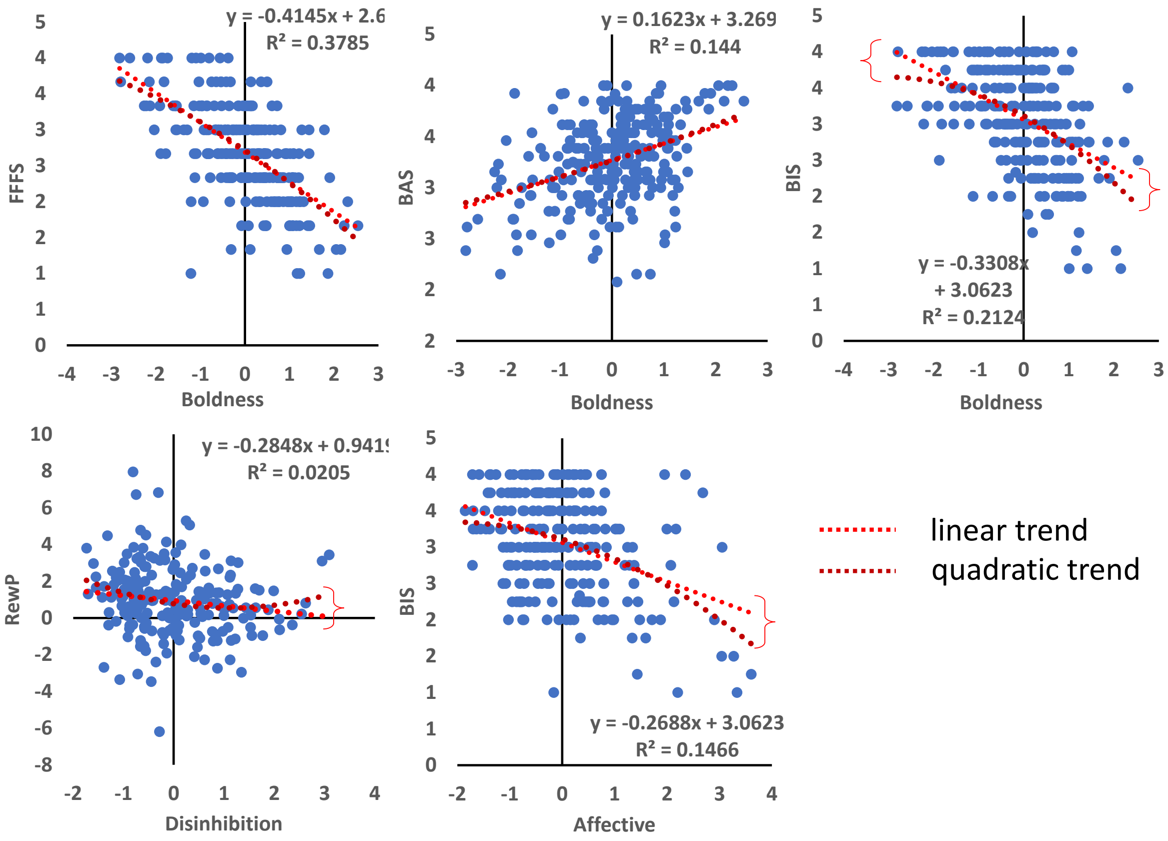

Note: Quadratic trends are shown for comparison with linear but did not produce significant improvement in the fit. BIS = Behavioural Inhibition System. FFFS = Fight, Flight, Freeze System. BAS = Behavioural Approach System. R^2 = square of zero order correlation coefficient (i.e. proportion of variance accounted for).
